# Mesoporous silica and vegetal extracts combined as sustainable stone heritage protection against biodeterioration

**DOI:** 10.1007/s00253-025-13475-5

**Published:** 2025-04-22

**Authors:** Andrea Campostrini, Agustí Sala-Luis, Pilar Bosch-Roig, Elena Ghedini, Michela Signoretto, Federica Menegazzo

**Affiliations:** 1 Instituto Universitario de Restauración del Patrimonio, Universitat Politècnica de València, Camí de Vera s/n, 46022 Valencia, Spain; 2 Catmat Lab, Department of Molecular Sciences and Nanosystems, Venice Ca’ Foscari University and INSTM Ruve, Via Torino 155, 30172 Venice, Italy

**Keywords:** Essential oils, Biodeterioration, Stone protection, Sustainable materials, Antifungal, Mesoporous MCM-41

## Abstract

**Abstract:**

Since biodeterioration is considered one of the main issues related to the conservation of cultural heritage stone materials, an investigation was conducted into preventive sustainable antimicrobial alternatives to protect the stone surfaces. The study focuses on using MCM-41 mesoporous silica particles and vegetal extracts: the mesoporous materials act as nanocontainers encapsulating the extracts, which instead serve as green antimicrobic compounds to inhibit microbiological proliferation. In this way, the antimicrobial features of the extracts are sustained for a more extended period, reducing the evaporation rate and diminishing the quantity required; the amount necessary to achieve the minimum inhibitory concentration was reduced due to the decrease in evaporation. Moreover, since the MCM-41 can host a higher quantity of product than is necessary to exert the antimicrobial effect, the duration of activity is further prolonged, releasing the extracts over time. Specifically, the mesoporous particles were impregnated with the vegetal extract of limonene and the essential oils of thyme and oregano. In vitro microbiological tests were conducted on two fungi (i.e., *Aspergillus tubingensis* and *Penicillium chrysogenum*), taken as model microorganisms from real-case scenarios. A combination of mesoporous silica and vegetal extracts was employed to develop a protective coating for stone surfaces, and tests were conducted on marble mock-ups. The promising synergic results show that this system could be of interest for preventing microbiological growth over stone surfaces, avoiding a visible aesthetic impact, being non-toxic for the environment or the operator, and preventing the extract from evaporating but holding it for a controlled release.

**Key points:**

• *Green antimicrobial system using porous silica as nanocontainer for plant extracts*

• *Encapsulated vegetal extracts to inhibit microbial growth on stone surfaces*

• *Stable and efficient coating against fungal species in vitro and on marble mock-up*

## Introduction

The degradation of stone materials represents a significant concern in preserving cultural heritage and architectural structures (Smith et al. 2008; De la Fuente et al. 2013; Morena et al. 2021; Elert and Rodriguez-Navarro 2022). Among the numerous factors contributing to this deterioration, microorganisms have emerged as a critical agent in the biodegradation process, both for outdoor and for indoor scenarios (Griffin et al. 1991; Di Carlo et al. 2022). Since biodeterioration is a critical issue in safeguarding Cultural Heritage materials, different techniques have been employed to limit or reduce it (Lo Schiavo et al. 2020; Scappin et al. 2022; Pinna 2022; Lamuraglia et al. 2023).

Microorganisms, such as fungi, play a fundamental role in the biodeterioration of stone materials; their ability to colonize and thrive on the surface of stones is helped by organic matter, moisture, and favorable environmental conditions (Balouiri et al. 2016; Stanaszek-Tomal 2020). As microorganisms proliferate, the morphology of stone surfaces undergoes profound alterations. Surface pitting, chromatic alterations, and loss of structural cohesion are common manifestations of microbial biodegradation (Randazzo et al. 2015; Liu et al. 2022). These alterations compromise the aesthetic and structural integrity of the stone materials, requiring extensive conservation measures. The study of the degradation of stone materials due to microbial activity represents a complex interplay of physico-chemical, biological, and environmental factors (Fernandes 2006; Michaelsen et al. 2013; Schröer et al. 2021). Addressing these challenges requires an interdisciplinary approach integrating microbiology, chemistry, environmental science, and conservation and restoration science.

The intervention usually undertaken to deal with microbiological colonization over stone materials is related to cleaning treatments with biocides (Gherardi et al. 2016; Franzoni et al. 2020; Gabriele et al. 2021). However, most of these products are toxic not only for the target organisms but also for the environment and the operator (Silva et al. 2020; Jones and Joshi 2021). This led to an increasing interest in using natural substances: in recent years, the application of vegetal extracts, such as essential oils, has garnered attention as a promising strategy for combating microbial deterioration (Macedo-Arantes et al. 2021; Cai et al. 2022; Ranaldi et al. 2022; Russo and Palla 2023; Santo et al. 2023; Sala-Luis et al. 2024).

Vegetal extracts can be derived from various plant sources and have long been employed for their diverse therapeutic properties. One of the notable advantages lies in their asserted fungicidal activity: their active molecules, such as terpenes and phenolic compounds, exhibit inhibitory effects on fungal growth and reproduction. They could, therefore, mitigate the risk of fungal colonization on various surfaces, including those of historical significance (Candela et al. 2019; Spada et al. 2021; Favero-Longo et al. 2022; Sanchis et al. 2023). This action against fungi places vegetal extracts as versatile agents for conserving cultural heritage materials vulnerable to microbial threats (Stupar et al. 2014; Granata et al. 2018; EL-Hefny et al. 2019; Antunes Filho et al. 2023).

Vegetal extracts such as essential oils (EOs) are also applied in the cultural heritage field, especially for cleaning treatments or prevention. The volatile antimicrobial molecules present in essential oils constitute a distinct and potent array of compounds.

Despite their features, using EOs in cultural heritage conservation is challenging. The concerns related to the potential alteration of artifact appearance, odor retention, operators’ exposures, and long-term stability require careful consideration in developing application protocols. Furthermore, the molecules with the highest antimicrobial action usually are the same and have high volatility; hence, the time efficacy of these products needs to be carefully considered. If volatility is not a problem for cleaning interventions since they can be trapped in cleaning poultices, it becomes an issue when dealing with preventive actions. To avoid evaporation, options for encapsulating EOs are proposed in the literature, such as embedding them in nanocarrier systems based on polymers (of different nature) or hydrogels. These systems have also been studied for applications in cultural heritage (Dresler et al. 2017; Fidanza and Caneva 2019; Zuena et al. 2021). Another advantage of encapsulating EOs is the reduction of exposure for operators. Although their toxicity is considered low to moderate, some components of EOs can be toxic in high amounts (Llana-Ruiz-Cabello et al. 2015; Dima and Dima 2015; Sala-Luis et al. 2024). Encapsulation can help reduce the possibility of direct contact with these substances for operators, thereby increasing worker safety (Lucia and Guzmán 2021; Fuentes et al. 2021).

Another promising way is to embed vegetal extracts in mesoporous matrices with a structure able to host substances, such as MCM- 41 mesoporous silica. These materials are widely used in various applications, including coatings for cultural heritage materials (Alfieri et al. 2017; Ruggiero et al. 2020a, 2020b; Bartoli et al. 2024).

Therefore, in this research, three vegetal extracts have been embedded in mesoporous SiO_2_ to study their antifungal preventive power. The investigated system aims to exploit the antimicrobial power of the extracts’ active molecules, avoiding evaporation and color-related issues, thanks to mesoporous silica’s morphological ability to store and later release the antimicrobial vegetal extracts. Specifically, MCM- 41 particles were employed to hold and release the vegetal extract of limonene and the essential oils of *Origanum compactum* and *Thymus vulgaris*.

The decision on which silica nanocontainer to focus on was based on the diameters of the nanopores, which are approximately 3 nm for MCM-41 and, for instance, 6 nm for SBA-15 (Thommes et al. 2002; Jana et al. 2004; Ramirez et al. 2012). Meanwhile, concerning the vegetal extracts, the choice fell on employing extracts, already tested by different authors in literature and employed in the conservation field, derived from plants highly diffused and endemic to the Mediterranean region in an optic of environmental sustainability and availability from Spain and Italy, the two countries partners in this work (Gakuubi et al. 2017; D’Agostino et al. 2021; Sparacello et al. 2021).

## Materials and methods

### MCM-41 synthesis

Mesoporous MCM-41 was synthesized using TEOS (Tetraethyl orthosilicate; CAS 78-10- 4) as a silica source and hexadecyltrimethylammonium bromide (CTAB; CAS 57-09- 0) as a template in NaOH (CAS 1310-73-2) basic solution. To reach a material with a pore diameter of ~ 4 nm, the mixture was continuously stirred at 300 rpm for 3 h at 25 °C and crystallized in a Teflon autoclave at 75 °C for 70 h, keeping pH = 11 (Campostrini et al. 2025). The mixture was then filtered, washed, dried at 25 °C for 20 h, and finally calcined in air (50 mL/min) at 510 °C (0.5 °C/min rate) for 6 h (Breda et al. 2006).

All the materials employed for this step were purchased from Sigma-Aldrich® (Merck Life Science S.r.l., Milan, Italy).

### Impregnation with vegetal extracts

MCM- 41 particles were impregnated, until the maximum of their absorption capability, by the incipient wetness impregnation (IWI) method with the vegetal extract of limonene and the essential oils of *Origanum compactum* and *Thymus vulgaris ct linalool*, leading to a concentration of ~ 1.6 mL g^−1^, once dried at RT. Briefly, a known amount of extract was poured on the silica powder and amalgamated with a glass spatula. In order to ensure optimal impregnation of the extract, a series of intermediate drying steps at RT were carried out.

The limonene extract was donated by Commercial Xúquer (S.A.R.L.), Valencia, Spain. The essential oils *Origanum compactum* and *Thymus vulgaris ct linalool* were purchased from Pranarôm International (S.A.), Ghislenghien, Belgium (BE). Limonene is a pure component present in different *Citrus* spp*.* EOs (Eddin et al. 2021), while oregano EO main component is carvacrol (Chouhan et al. 2017; Oliveira Ribeiro et al. 2020) and the main component of thyme EO is linalool, as indicated in the commercial product information (López et al. 2018; Díaz-Alonso et al. 2021).

### Microbiological sampling campaign

**Fig. 1:**
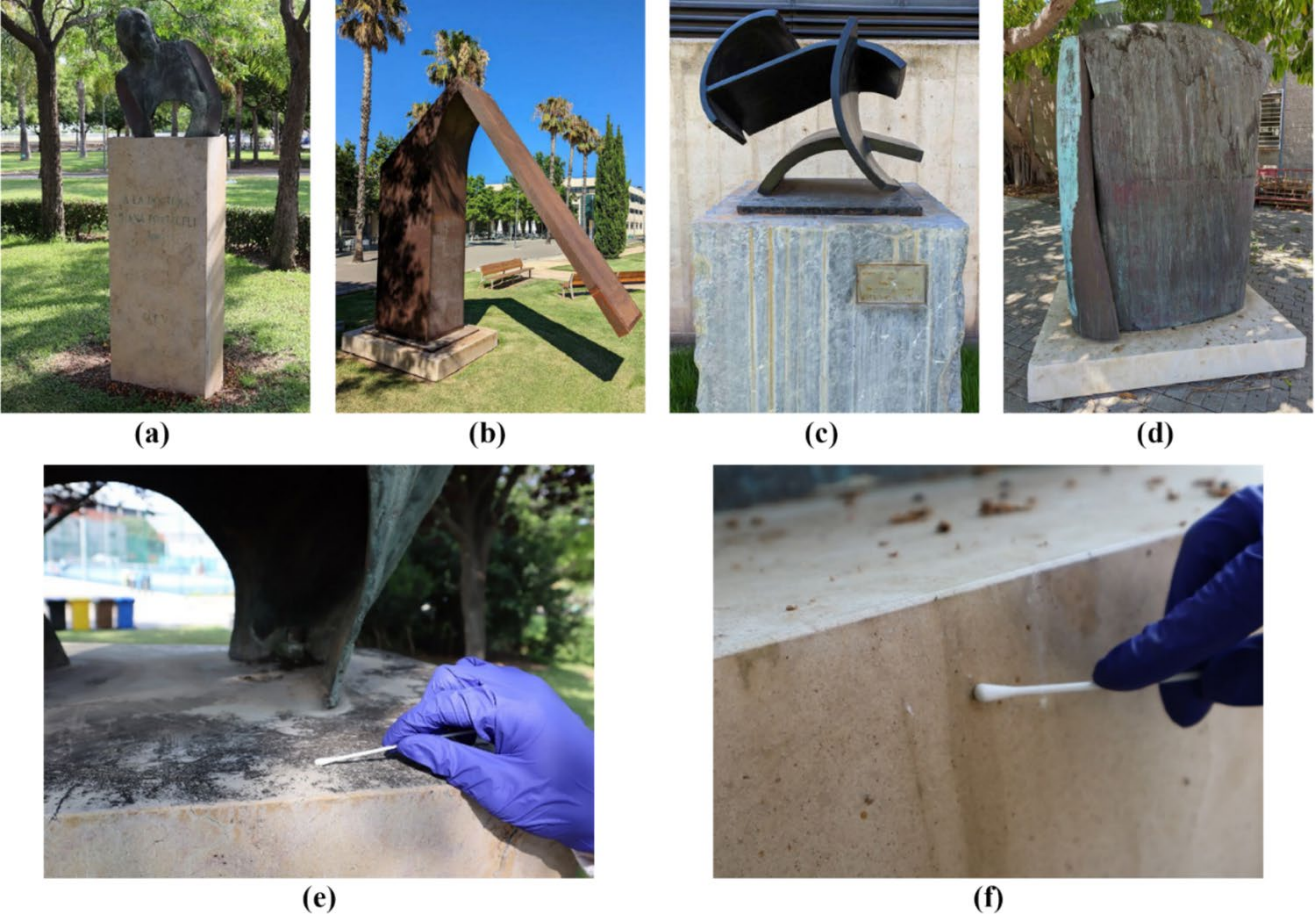
Statues from MUCAES- UPV: *Statue Homenaje a la Dra. Juana Portaceli* (**a**), *Statue Architectura del Silencio* (**b**), *Statue Homenaje a Manolo* (**c**), *Statue Torso H.* (**d**), and pictures of the sampling campaign (**e**,** f**)

To select the microorganisms to be used, sampling campaigns have been conducted on real-case-built heritage stone materials in Valencia, Spain. Precisely, as shown in Fig. 1, from marble parts of four statues from the Sculpture Museum of the *Universitat Politècnica de València*, *Vera Campus* (MUCAES-UPV): *Statue Homenaje a la Dra. Juana Portaceli*, *Statue Architectura del Silencio*, *Statue Homenaje a Manolo*, *Statue Torso H.* (1989–2007).

The samplings were executed by non-invasive methods, rubbing humid sterile cotton swabs on the stone surfaces (Silva et al. 2022). The samplings were plated on Petri dishes with Sabouraud Dextrose Agar (SDA, purchased from Sharlab, Barcelona, Spain). The attention was focused on fungi for their ability to produce pigments that could be absorbed by the stone porosity and lead to easthetical damage. The Petri dishes were incubated at 28 °C for 3 days for initial mycelium development and then monitored until the fungal colonies’ maturation (active and abundant sporulation).

### Fungal isolation and molecular identification

Based on the morphological characteristics of the fungal colonies obtained from the stones, the two most frequent fungi were selected and isolated for this study. The selected fungi were let grow in pure culture with an SDA cultivation medium in an incubator (Binder INC, Bohemia, EUA) at 28 °C for 3 days, reaching an optimal inoculation growth level with active sporulating conidiophores.

Genomic DNA was purified from the pure cultures and stored on the Whatman FTA™ Indicating Card for fungal isolates by STAB Vida Lda. (Portugal). Fragments of the fungal rDNA gene containing the ITS and D1/D2 regions were PCR amplificated using an ITS5/LR6 set of primers. FNA Sanger of the amplified products were aligned to generate consensus sequence and checked against the GenBank by BLAST of consensus sequence against NCBI database, applying a similarity score of ≥ 99.0%. Furthermore, the β-Tubulin genomic region has been sequenced and analyzed as the gene of choice for species-level differentiation within *Aspergillus* and *Penicillium* genera, especially when the ITS (Internal Transcribed Spacer) region sequence alone is insufficient as a single marker to distinguish closely related species.

First amplification and subsequent partial sequencing of the β-Tubulin gene (with bidirectional reads) were performed using the primers Bt2a (5′-GGTAACCAAATCGGTGCTGCTTTC- 3′) and Bt2b (5′-ACCCTCAGTGTAGTGACCCTTGGC- 3′) (Glass and Donaldson 1995). Then the sequences were compared using BLAST (Basic Local Alignment Search Tool) analysis (Altschul et al. 1997) against the NCBI public reference database (https://blast.ncbi.nlm.nih.gov/Blast.cgi). The percentages of similarity, the extent of overlapping fragments, and the names of microorganisms with the highest sequence identity were finally collected. Finally, the isolated strains used in this study were deposited in the CECT publicly accessible culture collection belonging to the WDCM.

### Minimal inhibition concentration determination by poisoned-food technique

The poisoned-food technique method was used to determine the minimal inhibition concentration (MIC). The method is based on mixing, within the cultivation medium, fungal growth inhibitors or potentially toxic substances (Balouiri et al. 2016). The medium was prepared by dissolving SDA powder in water (65 g/L) in the autoclave at 120 °C for 20 min in a flask and then poured into different falcon tubes. At this stage, different amounts of vegetal extract, *free* or encapsulated in the MCM- 41 SiO_2_, were added to the falcon tubes to reach the desired extract concentrations (from 0.05 to 5%—corresponding to ⁓0.45 to ⁓45 mg/mL). The *poisoned* cultivation media were poured into Petri dishes (Ø = 55 mm).

After medium solidification, a small circular inoculum (Ø = 6 mm) was cut from fresh, pure SDA fungal colonies and placed in the Petri disk’s center, making the colonized side of the circular inoculum touch the medium. They were hence incubated at 28 °C for 7 days, monitoring mycelium development: the presence of growth indicates that the tested product concentration does not inhibit the microorganism’s growth. Control tests were carried out with just SDA and with SDA with MCM- 41 mesoporous nanoparticles (without vegetal extracts). All tests were performed in triplicate. Pictures were taken after 3 and 7 days to evaluate the antimicrobial efficacy with a Canon EOS M50 camera. To establish the growth inhibition, the diameter of colonies was measured using ImageJ, then the percentage of inhibition was calculated, as reported in literature (Maung et al. 2021; Kahramanoğlu et al. 2022), using the formula (1):

1 $$Inhibition\,(\%)=\frac{\varnothing Control-\varnothing Test}{\varnothing Control}\times100$$

### Evaporation tests

To evaluate the volatility of the active molecules composing the three vegetal extracts, an evaporation test was carried out for each extract separately (Janatova et al. 2015): 100 µL of extract was placed in a Petri glass dish, keeping track of the weight loss over time with an analytical balance.

The test was repeated in the same conditions, with the best-performing encapsulated extract, to evaluate the decrease in volatility after impregnation. Therefore, 1 g of MCM-41 mesoporous SiO_2_ was impregnated with 1600 µL of extract and then weighted over time. The weight measure was carried out before starting the evaporation test (*T*_0_) and then repeated at different periods (*T*_*n*_), where *n* corresponded to every 3 min for the first hour, then every hour until 6 h, and later at 1–2–3–7–14–28 days. All data sets were processed with the software Origin 2021 by OriginLab, Northampton, MA, USA. The percentage of evaporation was determined using the formula (2):

2 $$evaporation\, (\%)=\frac{weight\, \, {T}_{n} }{weight\, {T}_{0} }\times 100$$

### Data analysis

Statistical analyses were conducted to summarize the data, including the calculation of means and standard deviations. All measurements and tests were performed in triplicate to ensure reliability and reproducibility. Outliers were identified and excluded to maintain data integrity. Microsoft Excel was used for data processing and computation to ensure accuracy and consistency in the results.

### Coating on marble mock-ups

To evaluate the applicative aim of the presented combination of vegetal extract and SiO_2_ MCM-41, the impregnated particles were dispersed in a 1:1 mixture of glycerol and 96% ethanol. This solution was then applied at a rate of 2 L/m^2^ on the upper face of sterilized white Carrara and green marble mock-ups.

The quantity of impregnated SiO_2_ was varied to develop two coating formulations, resulting in coverage levels of 0.7 or 7 g/m^2^ on 5 × 5 cm stone surfaces, corresponding to 0.1% (0.09 mg/mL) or 1% (0.9 mg/mL) of plant extract. These formulations were therefore named COT- 01 (0.1% vegetal extract and SiO_2_ MCM-41) and COT-1 (1% vegetal extract and SiO_2_ MCM-41). While the ratio of plant extract to SiO_2_ was maintained constant, the amount of impregnated SiO_2_ in the coating formulations was varied (17 mg/mL or 170 mg/mL).

### Microbiological test over coated marble mock-ups

To evaluate the antimicrobial activity of the treatment with the impregnated MCM-41 SiO_2_, the coated mock-ups were placed in some sterile cylindric recipients where SDA growth medium was poured until reaching the mock-up surface level. Once the medium was solidified, it was inoculated with fungal spores and covered the cylinder with a cotton lid to let oxygen pass through. The cylinders were incubated at 28 °C for 3 days for initial mycelium development and then monitored at room temperature (RT) until 60 days with a Canon EOS M50 camera.

## Results

### Microbiological sampling campaign and fungi identification

**Fig. 2:**
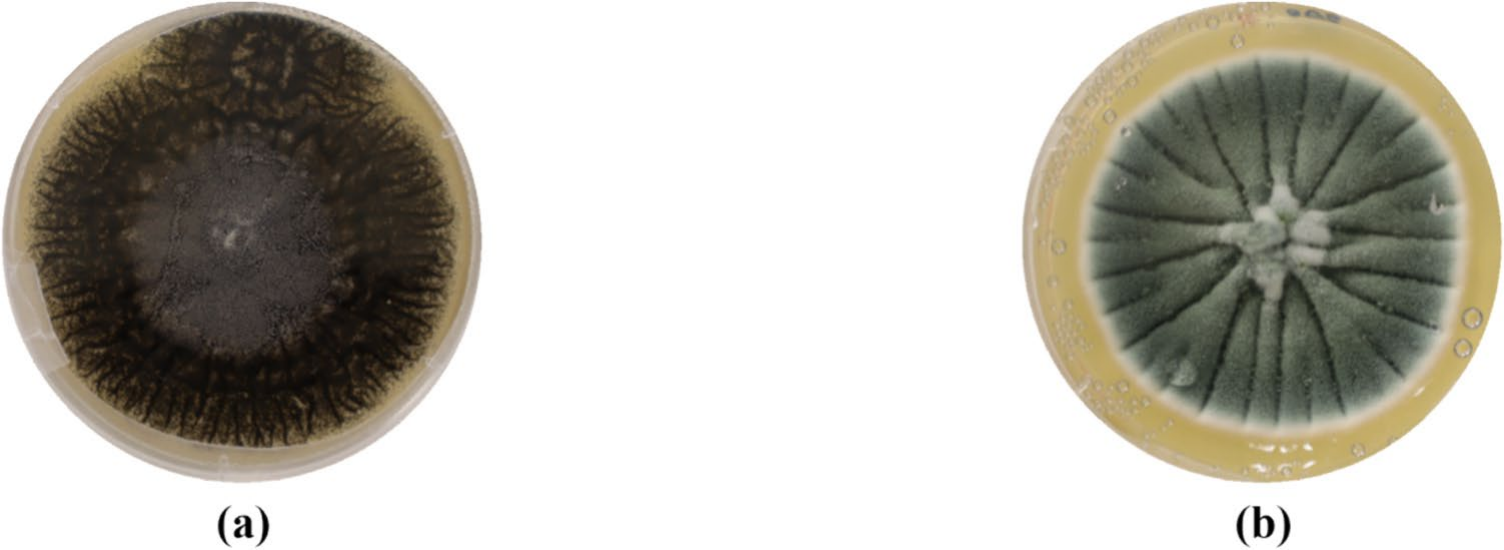
Pictures of the pure colonies of the two model microorganisms taken as reference for this study: *Aspergillus tubingensis* (CECT 21302) (**a**) and *Penicillium chrysogenum* (CECT 21303) (**b)**

The sampling campaign led to the selection of two fungi as model microorganisms, distinguished by their appearance: a black fungus (Fig. 2a) with white hyphae and black spores, and a green fungus (Fig. 2b) with white hyphae and green spores. Initially, morphological identification using optical microscopy classified them as *Aspergillus* sp. and *Penicillium* sp., respectively. Subsequently, molecular sequencing confirmed their species as *Aspergillus tubingensis* CECT 21302, showing 99.82% similarity (554/555 bp) with sequence HQ632707 (reference strain IHEM 13662) and 99.80% similarity (507/508 bp) with sequence EF661086 (type strain NRRL 4875), and *Penicillium chrysogenum* CECT 21303 exhibiting 100% similarity (477/477 bp) with sequence MZ078823 (strain 426E).

The selected and isolated microorganisms were molecular sequencing therefore identified as *Aspergillus tubingensis* (CECT 21302) and *Penicillium chrysogenum* (CECT 21303).

It is worth mentioning that both *Penicillium* sp. and *Aspergillus* sp. are two of the most common fungal genera found when dealing with the biodeterioration of stone surfaces; therefore, they have been selected for this study.

### Minimal inhibition concentration determination by poisoned-food technique

Minimal inhibition concentration (MIC) can be defined as the minimum amount of antimicrobial required to get a total inhibition. The poisoned-food technique determined MIC; first, *free* vegetal extracts were studied and, afterward, compared with their use encapsulated in the MCM-41 mesoporous silica matrix.

**Fig. 3:**
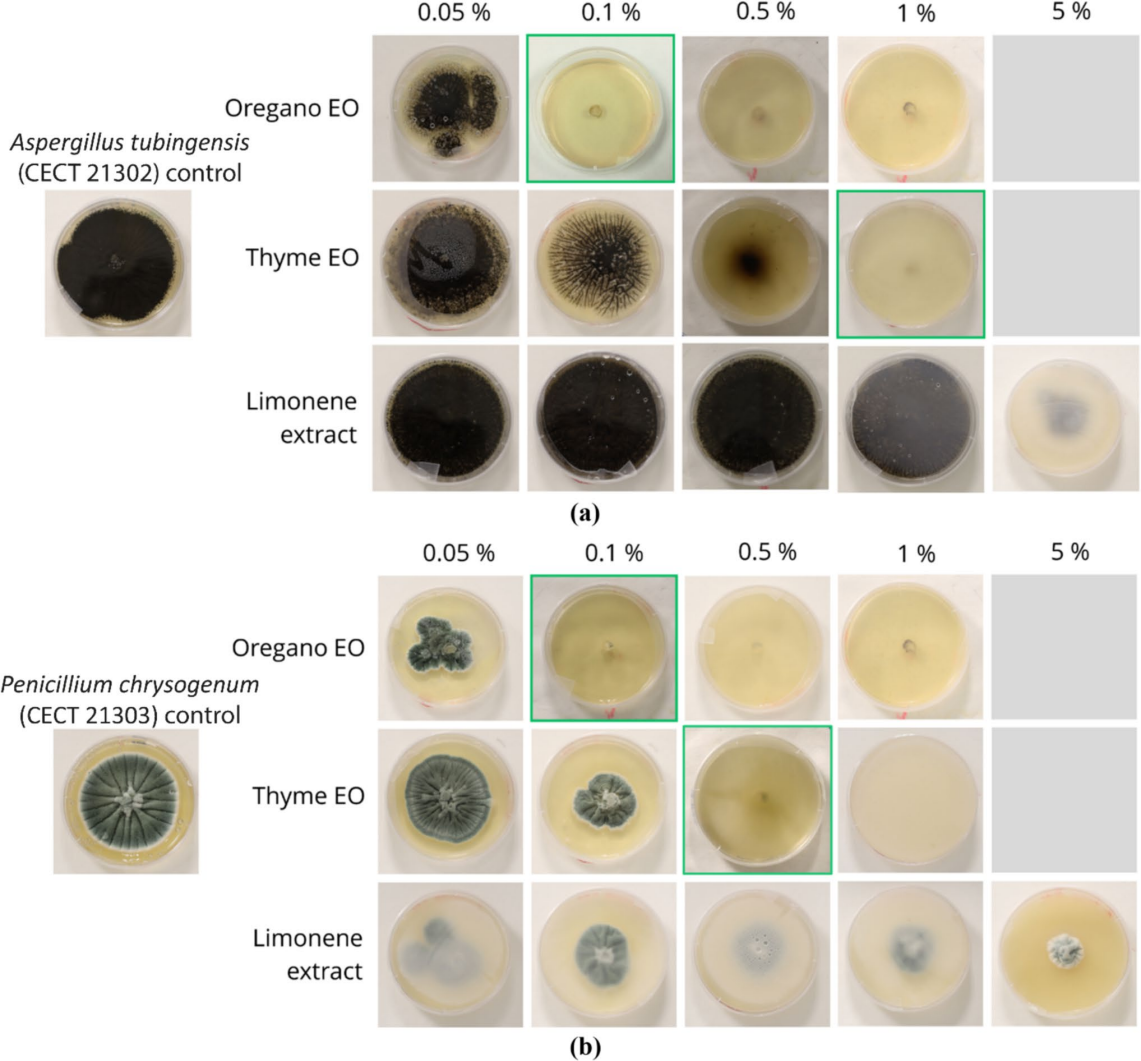
Pictures of the two reference fungi *A. tubingensis* (**a**) and *P. chrysogenum* (**b**) grown on an SDA medium with *free* vegetal extracts in different concentrations, following the poisoned-food technique. Highlighted in green is the relative minimum inhibitory concentration (MIC)

Figure 3 presents the pictures of the test results carried out on the *free* vegetal extracts, whereas Table 1 reports the relative average percentage of fungal inhibition, measured by the given formula.

**Table 1:** Percentage fungal inhibition of *A. tubingensis* and *P. chrysogenum* following the poisoned-food technique with *free* vegetal extracts

		Concentration of EO				
		0.05%	0.1%	0.5%	1%	5%
*A. tubingensis*	Oregano EO	29%	100%	100%	100%	nd
	Thyme EO	0%	11%	79%	100%	nd
	Limonene extract	0%	0%	0%	0%	84%
*P. chrysogenum*	Oregano EO	68%	100%	100%	100%	nd
	Thyme EO	33%	68%	100%	100%	nd
	Limonene extract	57%	68%	77%	79%	88%

The samples’ average SD was < 1.5%

*nd* no data

Concerning the results obtained with the *free* oregano essential oil (OEO) with both fungi, it was noticed that with a concentration of 0.1% or higher, there was no fungal development over the Petri dish. A test with a lower concentration of 0.05% was also conducted; in this case, mycelium development was instead noticed. The *free* oregano EO MIC was therefore set at 0.1%.

For what concerns *free* thyme EO (TEO), for the *A. tubingensis*, complete inhibition was reached at a higher concentration, equal to 1%. It can be observed that at a concentration of 0.5%, partial inhibition of the mycelium development is present. For *P. chrysogenum*, complete inhibition was set at 0.5%, and at 0.1%, partial inhibition was instead observed.

Partial inhibition might prevent a high spread of the microorganism over the surface but will not lead to an acceptable solution over time. The *free* thyme EO MIC was thus set at 0.5% for *P. chrysogenum* and 1% for *A. tubingensis*.

Finally, concerning the *free* limonene extract results, it was noticed that none of the concentrations previously studied successfully inhibited fungal growth. Therefore, the extract concentration was further increased to 5%. In this case, a delay in mycelium development was observed only for *A. tubingensis*. Nevertheless, it was insufficient to inhibit fungal growth totally. The concentration was not further increased since it would lead to a remarkably high amount of vegetal extract. Furthermore, it was possible to notice that the volatile components released by the limonene extract were degrading the Petri dishes’ plastic lids, making them permanently opaque.

**Fig. 4:**
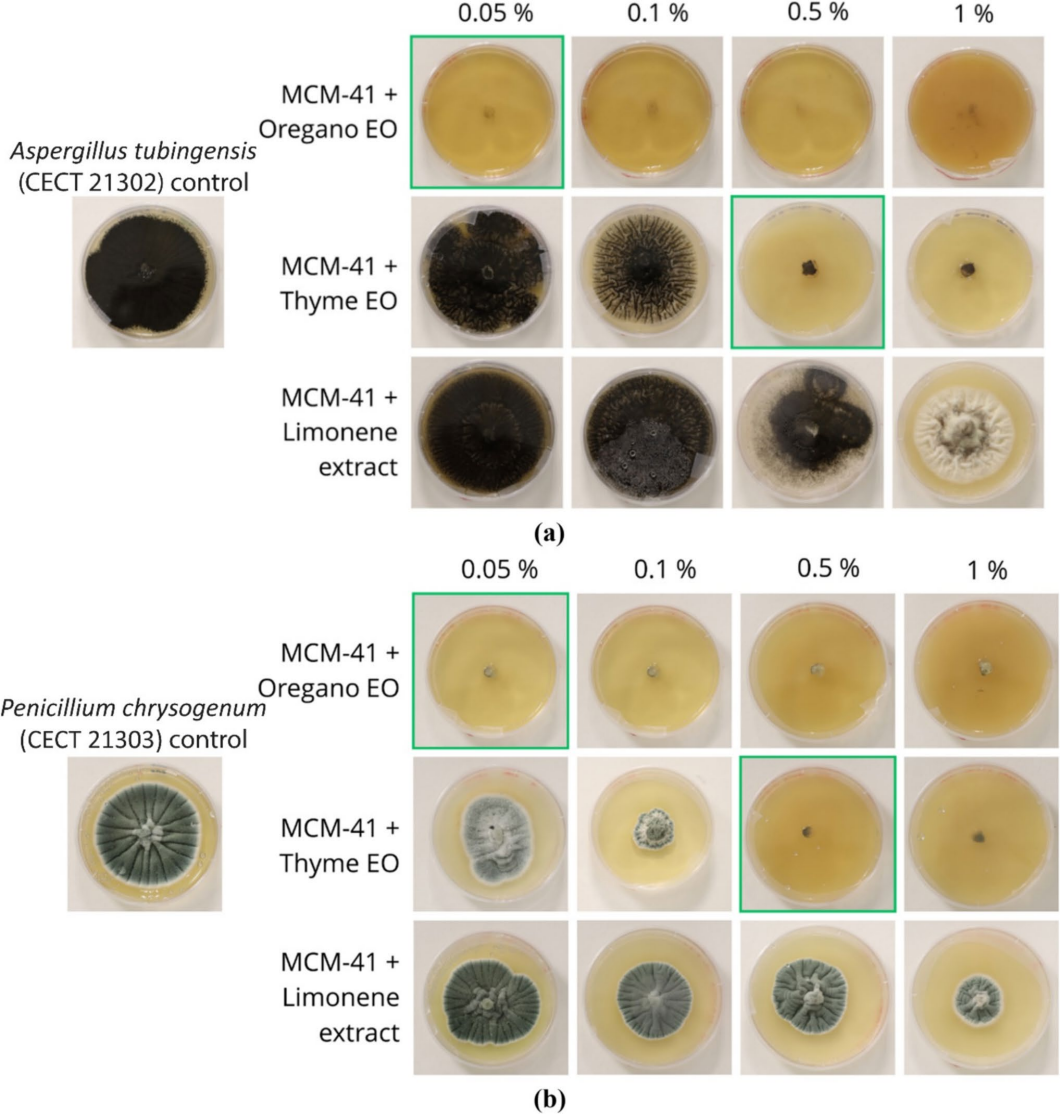
Pictures of the two reference fungi *A. tubingensis* (**a**) and *P. chrysogenum* (**b**) grown on an SDA medium with vegetal extracts encapsulated in the MCM- 41 SiO_2_ in different concentrations, following the poisoned-food technique. Highlighted in green is the relative minimum inhibitory concentration (MIC)

Figure 4 presents the test results on the vegetal extracts encapsulated in the MCM-41 mesoporous silica particles, whereas Table 2 reports the relative average percentage fungal inhibition.

**Table 2:** Percentage fungal inhibition of *A. tubingensis* and *P. chrysogenum* following the poisoned-food technique with *free* vegetal extracts

		Concentration of vegetal extract			
		0.05%	0.1%	0.5%	1%
*A. tubingensis*	MCM-41 + Oregano EO	100%	100%	100%	100%
	MCM-41 + Thyme EO	15%	29%	100%	100%
	MCM-41 + Limonene extract	0%	0%	13%	38%
*P. chrysogenum*	MCM-41 + Oregano EO	100%	100%	100%	100%
	MCM-41 + Thyme EO	58%	90%	100%	100%
	MCM-41 + Limonene extract	46%	63%	64%	83%

The samples’ average SD was < 1.5%

Regarding the encapsulated oregano EO, at the concentration of 0.1%, previously identified as MIC for the non-encapsulated system, no fungal development was observed for either *A. tubingensis* or *P. chrysogenum*. The concentration was therefore halved to 0.05%, which again did not let the fungi develop in the poisoned medium. Thus, the MIC for the encapsulated oregano EO was set at 0.05% for both fungi.

Equivalent results were obtained for the encapsulated thyme EO, for which fungal growth was not noticed at concentrations of 1%. Considering the halved concentration obtained with the encapsulated Oregano EO, an encapsulated thyme EO concentration of 0.5% was evaluated. This concentration was enough to totally inhibit mycelium development in the Petri for both fungi. Tests at the lower concentrations of 0.1% and 0.05% were also conducted; in this case, after 7 days, fungal growth was observed. Therefore, the encapsulated thyme EO MIC was set at 0.5% for both fungi.

Regarding the tests conducted with the particles holding the limonene extract, the fungal growth was visible in all the evaluated concentrations; therefore, it was not possible to determine the MIC neither for the encapsulated limonene extract. However, making a comparison with the results obtained from the *free* extract, it is possible to notice that for *A. tubingensis* (CECT 21302), at the concentration of 1%, the mycelium development and the spore formation are delayed in the encapsulated sample. Concerning instead the *P. chrysogenum* (CECT 21303), a reduction in the overall fungal development is visible proportionally to the increase of the extract concentration; another interesting detail is that the Petri dishes of the encapsulated sample are not opaque, suggesting a higher retention of the volatile molecules by the MCM-41 mesoporous particles.

The control tests with the two fungi growth in SDA alone or with SDA and MCM-41 mesoporous SiO_2_ without vegetal extract showed a complete Petri dish growth after 3–7 days incubation at 28 °C.

In summary, as described in Table 3, the MIC for oregano EO is 0.1% when *free* and 0.05% when encapsulated; the MIC for thyme EO is 0.5% both *free* and encapsulated; and finally, the MIC for limonene was not possible to be identified with the parameters employed in this study, neither when *free* nor when encapsulated.

**Table 3:** Minimal inhibition concentration (MIC) of the three vegetal extracts for the studied microorganisms

	*A. tubingensis* (CECT 21302)	*P. chrysogenum* (CECT 21303)
*Origanum compactum* EO	0.1%	0.1%
*Origanum compactum* EO + MCM- 41	0.05%	0.05%
*Thymus vulgaris ct linalool* EO	1%	0.5%
*Thymus vulgaris ct linalool* EO + MCM- 41	0.5%	0.5%
Limonene extract	> 5%	> 5%
Limonene extract + MCM- 41	> 1%	> 1%

Overall, considering the in vitro results and the obtained MIC, the best performing vegetal extract resulted to be *Origanum compactum* EO, encapsulated in the mesoporous MCM-41 SiO_2_ which showed very promising results and was therefore selected for the test on marble mock-up surfaces.

### Evaporation tests

**Fig. 5:**
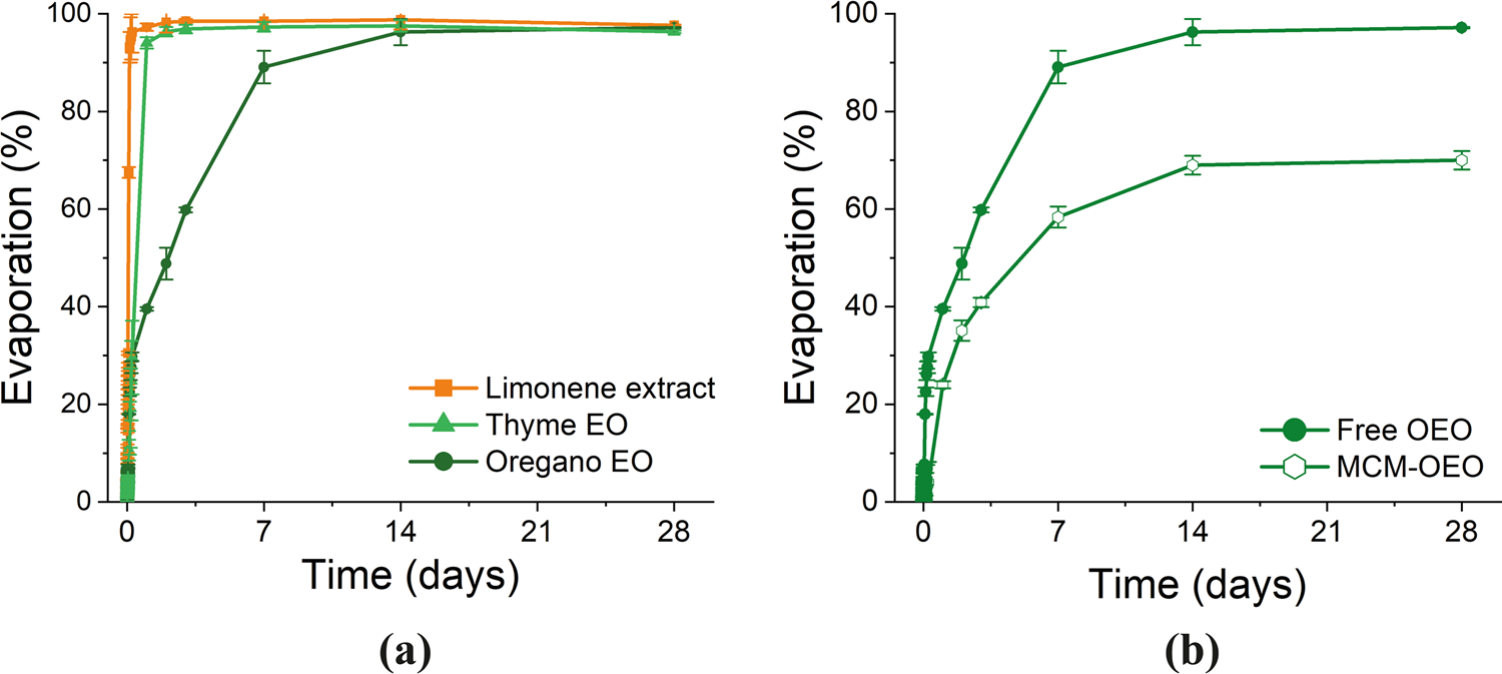
Graph reporting the evaporation curves of the three *free* vegetal extracts (**a**) and comparison between the evaporation curve of *free* and MCM-41 SiO_2_ encapsulated oregano essential oil (**b**)

Figure 5a shows the evaporation curves of the *free* vegetal extracts over 28 days. It can be noticed that limonene extract and thyme EO have a similar evaporation trend; within the first hours, they both almost reach complete evaporation, whereas *free* oregano EO has a slower trend, going beyond 95% after around 14 days and reaching 100% after 28 days. In Fig. 5b, the curves of the *free* oregano EO (Free OEO) are compared with the encapsulated in the mesoporous MCM- 41 SiO_2_ (MCM-OEO); it can be noticed that the oregano EO evaporation is reduced by around 30% after 28 days, and the mesopores of MCM- 41 can indeed trap the volatile molecules of the EO and prolongate the evaporation time.

### Microbiological test on coated marble mock-ups

**Fig. 6:**
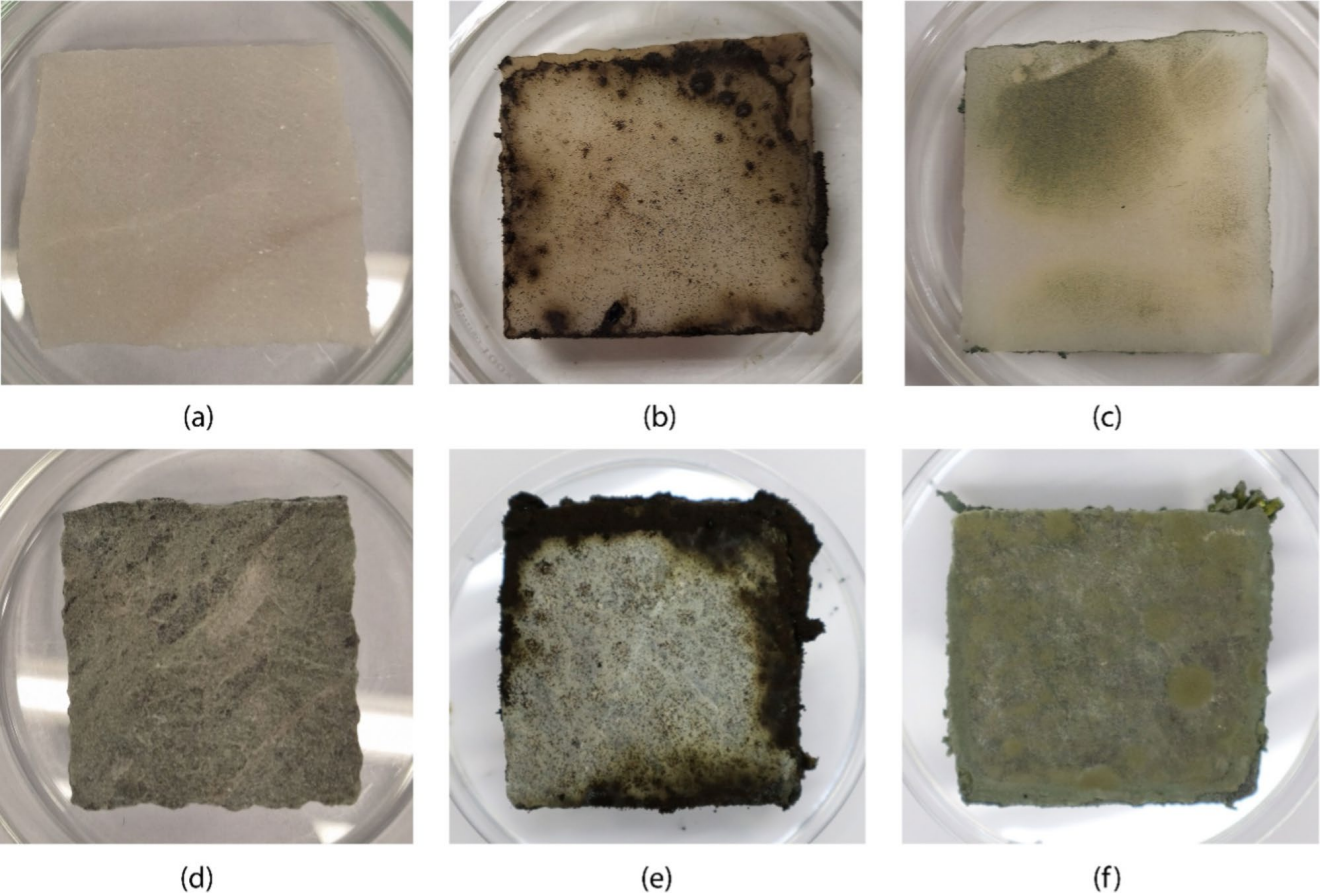
Pictures of the white marble mock-ups (**a**, **b**, **c**) and the green marble mock-ups (**d**, **e**, **f**) before (**a**, **d**) and after being colonized by the fungi *A. tubingensis* (**b**, **e**) and *P. chrysogenum* (**c**, **f**). Pictures were taken after 1 month from inoculation

The fungal growth on the uncoated marble mock-ups was abundant for both fungi, presenting mycelium in the vegetative state expanding over the surface. Figure 6 illustrates this phenomenon, particularly visible in the white marble mock-ups.

For clarity, the results obtained from the mock-ups treated with the two coatings (i.e., COT-01 and COT-1), containing MCM-41 mesoporous SiO_2_ and *Origanum compactum* EO, will be reported starting from those of COT-01 (with 0.1% EO) and following with those of COT-1 (with 1% EO).

**Fig. 7:**
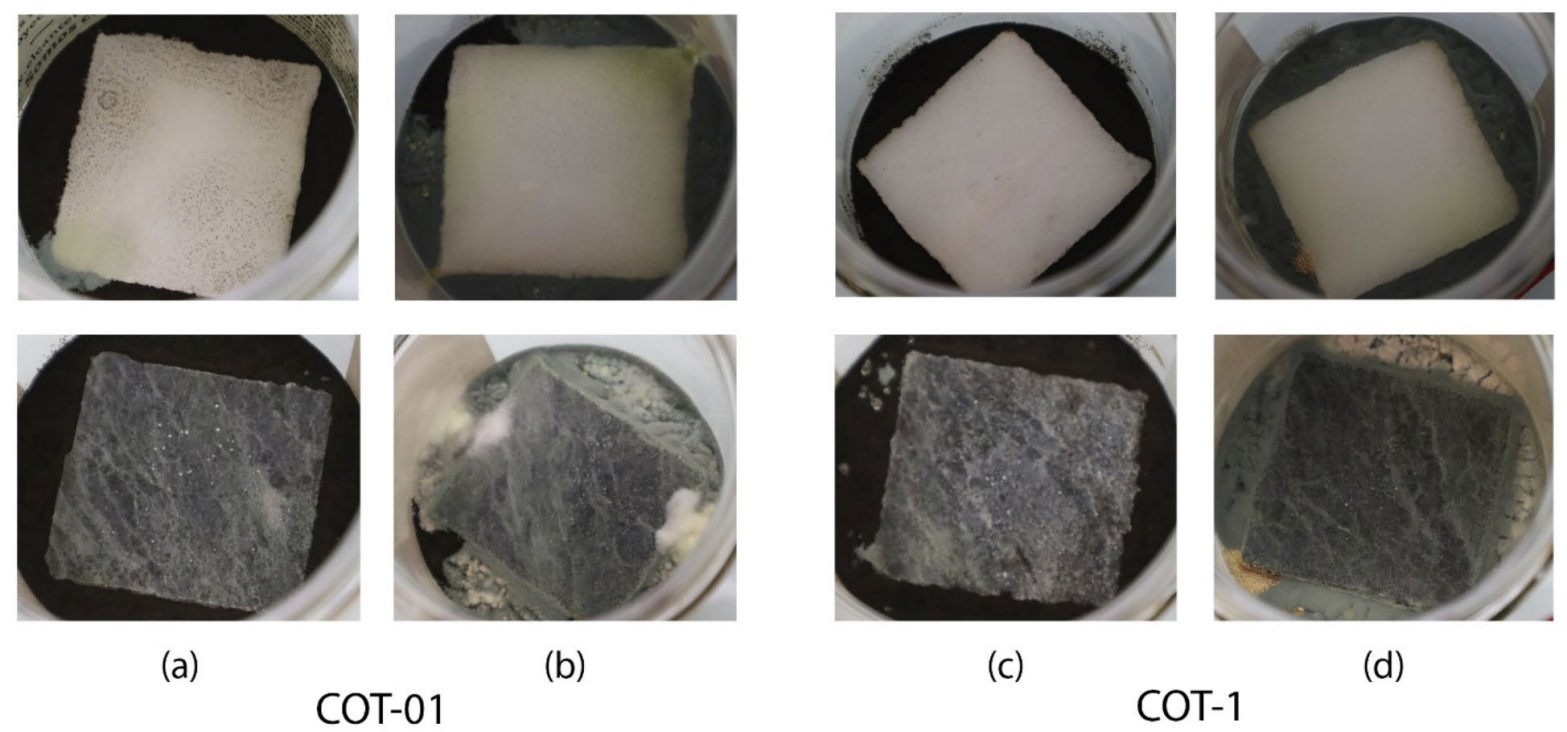
The white Carrara marble (upper) and green marble (lower) mock-ups treated with the coating COT-01 and further colonization by *A. tubingensis* (**a**) and *P. chrysogenum* (**b**); and the white Carrara marble and green marble mock-ups treated with COT-1 and further colonization by *A. tubingensis* (**c**), and *P. chrysogenum* (**d**); pictures were taken after 60 days

The test conducted with the *A. tubingensis* showed that, after 2 months, the surfaces treated with COT-01 were partially colonized by the fungi (Fig. 7b), particularly visible on the white marble, where dark little dots on the surface, likely spores in a germination state, were observed. Comparable results were observed with *P. chrysogenum*, where the COT-01 coating was once again insufficient to inhibit fungal growth (Fig. 7c), particularly visible on the green marble mock-up where the white hyphae had no trouble colonizing the surface. It is also interesting to notice that the *free* oregano EO, according to the evaporation curves, has fully evaporated in 1 month. However, when encapsulated, it persists for a longer period, at least twice as long, according to these tests conducted on the samples over 2 months.

On the other hand, the COT-1 coating, which contained more EO-impregnated MCM-41, was more effective in preventing fungal colonization. Specifically, regarding *A. tubingensis*, it can be observed that the fungi are confined on the agar on the side of the mock-up (Fig. 7e). As for *P. chrysogenum*, there is an overall reduction in colonization on the surface (Fig. 7f); however, a greenish patina can be seen on the sides of the mock-ups, especially on the white marble one.

## Discussion

The antimicrobial inhibitory potential of three plant extracts (limonene, *Origanum compactum,* and *Thymus vulgaris*) was evaluated when *free* or encapsulated in mesoporous MCM-41 silica (SiO_2_), determining the plant extracts evaporation rate and MIC against two fungal strains (*Aspergillus tubingensis* and *Penicillium chrysogenum*) isolated from real-case stone heritage materials.

The results indicated that encapsulation of the extracts led to a reduction in the MIC or the general growth of the fungi; it is, in fact, known from the literature that encapsulating such vegetal extract can increase its antimicrobial activity (El Asbahani et al. 2015; Ribeiro-Santos et al. 2017; Sundar and Parikh 2023; Lobato-Guarnido et al. 2023). Specifically, embedding the oregano essential oil into the MCM-41 mesoporous SiO_2_, a halving in the MIC of the studied fungi, was noticed. Overall, oregano essential oil exhibited the most favorable performance at the lowest concentration, thanks to its established antimicrobial ability (Plati and Paraskevopoulou 2022; Milagres de Almeida et al. 2023). Subsequently, an evaporation test on the MCM-41 impregnated with oregano EO was conducted, and the result was a reduction in the evaporation time. The outcomes suggest a correlation between volatility and antifungal activity: it is known that volatile molecules contribute to the antimicrobial efficacy of the extracts (Sanchis et al. 2023; Yammine et al. 2024). Therefore, encapsulation within the silica matrix diminishes evaporation and enhances the antimicrobial activity, suggesting prolonged efficacy over time, as already mentioned in literature in other fields of application (Beirão da Costa et al. 2012; Rodriguez-Garcia et al. 2016; El Abdali et al. 2023). Indeed, the *free* oregano EO showed the slowest evaporation rate, further diminishing when encapsulated. This phenomenon can be easily linked to the MCM- 41 silica ability to physically hold the active compounds into its ordered porous structure and later gradually release them (Campostrini et al. 2025).

Consequently, oregano EO encapsulated in mesoporous MCM-41 silica (SiO_2_) was subsequently tested on white and green marble mock-ups as the principal antifungal preventive component of coatings treatments at two concentrations (0.1% and 1%).

In the in vitro test, the growth medium was treated with the extracts, effectively inhibiting fungal growth. In contrast, for the test on marble mock-ups, only Sabouraud Dextrose Agar (SDA) was applied around the mock-ups, providing a fertile environment for fungal proliferation. Therefore, the quantity of EO-encapsulated MCM-41 in the coatings was adjusted to achieve an oregano EO concentration matching the MIC of *free* oregano (0.1%) and a higher concentration (1%).

Therefore, two coating bases were developed, COT- 01 and COT- 1, depending on the concentration of MCM-41 impregnated with oregano EO corresponding to 0.1% and 1% of *free* oregano EO, starting from a coating base made of a mixture of glycerol and ethanol, as previously studied by the authors (Lamuraglia et al. 2023).

The coating COT-01 was insufficient to inhibit fungal growth, whereas the coating COT-1 showed better efficacy in limiting microbiological proliferation. This disparity can be attributed to the distribution and concentration of MCM-41 particles within the coatings. In COT-01, the SiO_2_ particles may need to be adequately spaced or uniformly dispersed across the surface. Conversely, the COT-1 coating contained more impregnated MCM- 41, resulting in elevated antimicrobial activity. Overall, the quantity of SiO_2_ employed in COT-1 proved sufficient to limit fungal proliferation on the marble surfaces.

The difference between in vitro (MIC 0.05%) and “in mock-up” results (MIC 1%) can be explained by underlining the difference in the two systems: in the in vitro test, the impregnated SiO_2_ was directly dispersed in the cultivation medium where a 6-mm diameter fungal inoculum was physically placed, whereas in the “in mock-up test” the impregnated SiO_2_ used in the coatings needed to inhibit the growth of a higher amount of fungal colonies, since the cultivation medium around the stone mock-up was not “poisoned” and therefore the fungi had no problem in proliferating there.

This study highlights the potential of mesoporous MCM-41 silica encapsulating vegetal extracts as a sustainable and effective solution for inhibiting microbiological growth on cultural heritage stone surfaces. Impregnating these mesoporous particles with limonene, thyme, and oregano extracts reduced the MIC required to prevent fungal proliferation, indicating enhanced antimicrobial efficacy. Moreover, the encapsulation process mitigated evaporation rates, prolonging the duration of antimicrobial activity while minimizing the quantity of extract needed (Mutlu-Ingok et al. 2020; Mukurumbira et al. 2022). The results with oregano essential oil (EO) were particularly promising, demonstrating the most favorable performance regarding antimicrobial activity and evaporation rate reduction.

Further tests conducted on marble mock-ups, similarly shown in literature by Ramadan S. (Ramadan et al. 2024), confirmed the efficacy of MCM-41 mesoporous SiO_2_-encapsulated oregano EO as a component of the base of the protective coatings. The coating containing 1% oregano EO (COT-1) exhibited superior antimicrobial activity compared to 0.1% (COT-01). The disparity in effectiveness between coatings with different concentrations can be attributed to variations in distribution, the quantity of impregnated particles, and the related quantity of plant extract. Coatings with a higher concentration of impregnated MCM-41 demonstrated better antimicrobial performance, underlying the importance of uniform dispersion across the surface.

The findings suggest that the combination of MCM-41 mesoporous SiO_2_ and vegetal extracts holds significant promise for inhibiting microbiological growth on stone surfaces, offering a sustainable alternative to traditional antimicrobial treatments. Even though mesoporous materials technology is highly studied in other fields, such as the pharmaceutical one (Monfared et al. 2023; Alyami et al. 2023; Rani et al. 2024), its employment in combination with natural extracts and its application in conservation science is still a novelty. In fact, in the literature, a lot of recent works related to the use of essential oils for their antimicrobial features can be found. Nevertheless, the field of application, the aim, or the release system employed (if present) is different. A.P. Santo (Santo et al. 2023), in their paper, used essential oils to contrast biodeterioration on marble, showing how EOs can be employed as sustainable biocides with similar efficiency in comparison with traditional products. However, the EOs are employed for cleaning interventions: the antimicrobial action is not aimed to last for a long period. Other authors have also studied similar applications, such as M. Spada (Spada et al. 2021) or R. Candela (Candela et al. 2019). The encapsulation of EOs is presented by G. Granata (Granata et al. 2018), M. Kapustová (Kapustová et al. 2021), or F. Plati (Plati and Paraskevopoulou 2022) employing polymer-based nanocapsules to host essential oils, for application mainly in the alimentary field. Meanwhile, I. Romano (Romano et al. 2020) presents the use of polymer-encapsulated EOs in the field of stone cultural heritage. However, the latter nanostructured materials are totally different from the one presented in this study since they are not aimed for a long-term release apart from their different chemical and physical characteristics. The same can be said for the study presented by R. Ranaldi (Ranaldi et al. 2022), which employed EOs in hydrogel suspensions for the restoration of stone cultural heritage.

This approach addresses the critical issue of biodeterioration in cultural heritage conservation, which, as previously described, is one of the main issues to deal with in a restoration intervention (Ranalli et al. 2018) and presents opportunities for broader applications in architectural preservation and restoration efforts. This study focused on preventing the deterioration caused by fungi in vitro and in mock-ups submitted into an extreme environmental condition (high temperature and humidity). Further studies must be done to test this product in more real situations (with complex biofilms), on environmental conditions, and for longer periods. Furthermore, research must be done for optimizing the coating formulation and application techniques, as well as the in-deep evaluation of the leak of alterations on the treated stone surfaces; all this will be considered for future steps of this work.

## Data Availability

The authors declare that the data supporting the findings of this study are available within the paper. Other eventual supplementary information is available from the authors on reasonable request.
